# Colistimethate Acidic Hydrolysis Revisited: Arrhenius Equation Modeling Using UPLC-QToF MS

**DOI:** 10.3390/molecules26020447

**Published:** 2021-01-16

**Authors:** Ioanna Dagla, Anthony Tsarbopoulos, Evagelos Gikas

**Affiliations:** 1 Laboratory of Pharmaceutical Analysis, Division of Pharmaceutical Chemistry, Faculty of Pharmacy, School of Health Sciences, National and Kapodistrian University of Athens, Panepistiomiopolis, Zografou, 157 71 Athens, Greece; idagla@pharm.uoa.gr; 2 Laboratory of Pharmacology, Department of Descriptive-Functional Studies, Medical School, National and Kapodistrian University of Athens, 115 27 Athens, Greece; atsarbop@med.uoa.gr; 3 Laboratory of Analytical chemistry, School of Chemistry, National and Kapodistrian University of Athens, Panepistiomiopolis, Zografou, 157 71 Athens, Greece

**Keywords:** colistimethate, stability, Arrhenius, assay, LC-MS

## Abstract

Colistimethate (CMS), the prodrug of polymyxin E (colistin), is an antibiotic widely used as a last-line therapy against multidrug resistant Gram-negative bacteria, but little is known about its pharmacokinetics as its administration has stopped as a result of high neuro- and nephro-toxicity. The measurement of CMS levels in patients’ biological fluids is of great importance in order to find the optimal dose regimen reducing the drug toxicity. Until now, CMS assay methods are based on the indirect determination after its hydrolysis to colistin (CS). Herein, the aim is to find the optimal conditions for the complete hydrolysis of CMS to CS. The reaction was studied at accelerated conditions: 40 °C, 50 °C, and 60 °C, and the results were evaluated by assessing the Arrhenius equation and computation employing the Tenua software. A validated analytical methodology based on ultra-performance liquid chromatography (UPLC) coupled to a hybrid quadrupole time of flight (QToF) instrument is developed for the simultaneous measurement of CMS and CS. The current methodology resulted in complete hydrolysis, in contrast with the previously reported one.

## 1. Introduction

Colistin (CS) is an antibiotic, used for the treatment of multidrug-resistant infections caused by Gram (-) bacteria. Despite its neuro- and nephro- toxicity, it is widely used as a last resort antibiotic against multidrug (MDR)- and extensively drug-resistant (XDR) strains, which are resistant to at least three and almost all antimicrobial classes, respectively [1,2]. CS is a cyclic lipopeptide as its structure encompasses a decapeptide consisting of l-α-γ-diaminobutyric (Dab), leucine (Leu), and threonine (Thr); seven of the aminoacids forming a ring; and a fatty acid moiety attached to the *N*-terminus. The drug is produced by the *Bacillus polymyxa* var. *Colistinus* as a very complex mixture of structurally related compounds differing from each other to the fatty acid moiety, with two of them, namely CSA and CSB, being dominant. The two main forms of CS differ only by a methylene group; CSA has a 6-methyloctanoic, whereas CSB has a 6-methyleptanoic acid [3,4].

CS is administrated either as colistin sulfate for oral and topical use [5] or as colistimethate sodium (CMS) for aerosol inhalation or for i.m. or i.v. injection [6]. CMS is the prodrug of CS, produced by sulfomethylation of the five free primary amine groups of CS, affording the active form after in vivo hydrolysis. However, the structure of CMS is unknown, as components with a different degree of sulfomethylation and multiplicity of substitution of amine groups (mono and double sulfomethylated) result after the reaction. Inconsistencies of CMS content have been observed among different batches [7]. It is worth mentioning that these variations lead to different plasma concentration of the formed active colistin influencing the bioavailability and the dose regimen of CMS [8,9].

Several analytical methods have been developed for the measurement of CS and CMS levels in various biological materials, as CS assay is crucial for shedding light in its pharmacokinetics and dosage regimen in order to eliminate the drug toxicity [10,11]. Although some developed methods include high performance liquid chromatography (HPLC) coupled to fluorescence detector [12,13,14], the majority are based on hyphenated mass spectrometry (MS) techniques [15,16,17,18]. Measurement of the prodrug, i.e., the CMS levels, is also important for pharmacokinetic studies, but its assessment is performed indirectly after acidic hydrolysis to CS, as the precise CMS structure and, consequently, its mass are unknown. Various hydrolysis methods have been reported, by the addition of 0.5 or 1 M sulfuric acid employing a reaction duration of 10 to 60 min at room temperature. Table 1 summarizes the methods that have been reported for the hydrolysis of CMS, which were based on the methodology developed by Li et al. [19]. The authors used FMOC-Cl fluorescence derivatization. Because of the fact that every free amino group can be derivatized and taking into account the heterogeneity of the molecule (i.e., the fact that CMS has natively free amino groups in partially sulphomethylated derivatives or free amino groups can result by partial CMS hydrolysis), the FMOC-Cl derivatization method could erroneously lead to fluorescence signals for other molecular species, besides CSA and CSB.

The aim of the current work is the study of CMS hydrolysis to CS in the presence of sulfuric acid, in order to model the reaction and calculate the Arrhenius equation and focus on the establishment of proper conditions for the complete hydrolysis of CMS to CS. To study the hydrolysis kinetics, a validated analytical methodology based on ultra-performance liquid chromatography–mass spectrometry (UPLC-MS) was developed for the simultaneous measurement of CS and CMS.

## 2. Results and Discussion

### 2.1. LC-MS

Colistin is considered as one of the last resort antibiotics against the “superbugs”. Despite the fact that it has been in clinical use for more than 50 years, little is known about its pharmacokinetics and the dose regimen that should be obeyed in order to reduce its neuro- and nephro-toxicity. Several analytical methods have been developed for exploring its pharmacokinetics, by measuring the CMS and CS levels in biological fluids [23,24,25]. However, no analytical method has been reported for the direct quantitation of CMS in plasma; instead, the assay is performed after acidic hydrolysis of CMS to CS. Thus, complete hydrolysis or at least the precise knowledge of the reaction extent is a crucial perquisite for the reliable determination of CMS levels in biological material. In the present work, the kinetics of CMS hydrolysis by addition of sulfuric acid were studied using a validated method for measuring directly the CMS. Because of the complexity of CMS per se, it was selected to perform the hydrolysis modeling in water followed by the application of the method in plasma in order to verify the results.

In the first step, an analytical method based on UPLC-MS was developed for the quantitation of CS and CMS. Infusion of CS and CMS at a concentration of 10 μg mL^−1^ examining both negative and positive ionization modes was performed. The compounds presented higher ionization efficiency in positive electrospray ionization (ESI), affording singly protonated [M + H]^+^ ion peaks at *m*/*z* 1169.74 for CSA and 1155.74 for CSB. However, CMS was not represented as a single ion peak, but rather as a plethora of signals, ranging from *m*/*z* 1147.73 to 1471.77, reflecting the complexity of CMS (various substituted derivatives differing to the degree of amine substitution) peaks corresponding to source-induced dissociation. Thus, a full scan mode was selected for the detection of CS and CMS over the range of *m*/*z* 1000–1500.

The chromatography was optimized in order to facilitate the separation between the two forms of CS, i.e., CSA and CSB, and that of CMS. Under the chromatographic conditions described above, CMS eluted as one peak, despite the fact that it is actually a very complex mixture. This was justified because the goal of the current work was to develop a measurement methodology of the total CMS content in pharmaceutical products and study its acidic degradation, and not to find the differences on the basis of its components, as has been previously described by our laboratory [7].

**Figure 1:**
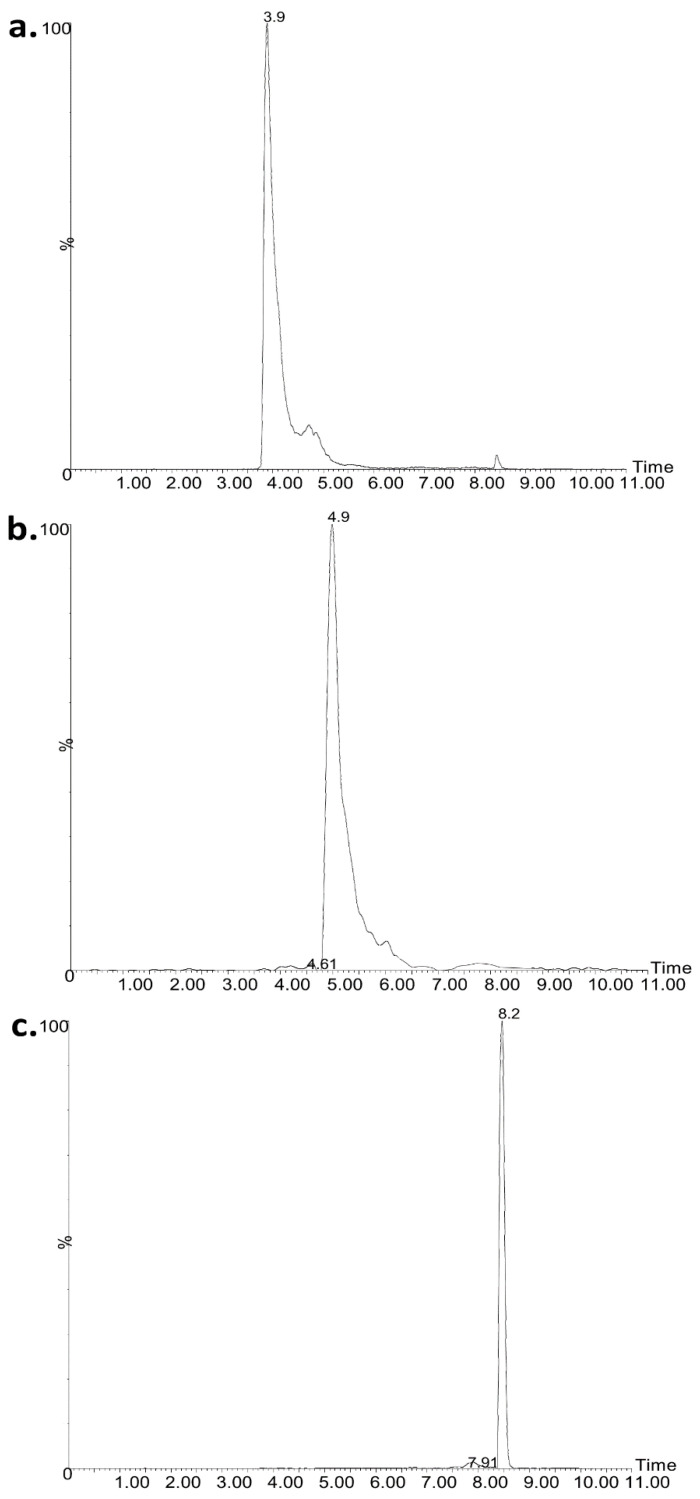
Representative ultra-performance liquid chromatography coupled to mass spectrometry (UPLC–MS) chromatogram of (**a**) CSB at 3.9 min, (**b**) CSA at 4.9 min, and (**c**) CMS at 8.2 min at concentration of 15.378, 6.622, and 22.0 μg mL^−1^, respectively. CS, colistin; CMS, colistimethate sodium.

The peaks were integrated using QuanLynx as implemented to the Masslynx suite. For the integration, the singly charged ions were employed either as protonated or as their corresponding sodium adducts. The *m*/*z* values of the quantifier ions employed were as follows: 1169.74 and 1191.74 for CSA; 1155.74 and 1177.74 for CSB; and 1193.76, 1205.77, 1217.77, 1215.77, 1227.75, 1239.75, 1251.75, 1331.69, and 1343.67 for CMS. A typical chromatogram is presented in Figure 1.

### 2.2. Method Validation

The method was developed for studying the acidic hydrolysis, but it was realized that it could also be used for assessing the content of CMS products. The injectable form of CMS was employed for the development of the methodology. Thus, each injectable CMS vial contains 1 MIU and, according to EMA [26], is equal to 80 mg of CMS and 34 mg of colistin base activity (CBA). This activity to mass equivalence was used for the calculations hereafter.

#### 2.2.1. Quantitation of the Ratio of CSA and CSB in Reference Sample

Determination of the stoichiometry of the two forms of CS in the reference sample was performed, because slightly different ratios were observed depending on the batch. Applying the developed methodology to the analysis of the CS reference sample (*n* = 5), it was observed that the content of CSA and CSB in the reference sample was 30.1 (±0.9)% and 69.9 (±0.5)%, respectively.

#### 2.2.2. Standard Curves

The content was estimated from the calibration curves, ranging from 1.806 to 6.622 μg mL^−1^ for CSA, from 4.194 to 15.378 μg mL^−1^ for CSB, and from 2 to 22 μg mL^−1^ for CMS. These ranges were suitable for the study of acidic hydrolysis of CMS as well as for the CMS quantitation in pharmaceutical products.

Linear regression models were used for CSA and CSB, whereas a quadratic regression model was selected for CMS. Three standard curves were run and analyzed, and the independent fits from each curve were compared with a global fit, using the extra sum-of-squares F test as implemented in GraphPad Prism 8.0, in order to find whether the best-fit values of the slope and the intercept differed between the data sets. The *p*-values (*p* = 0.56 for CSA, *p* = 0.52 for CSB, and *p* = 0.47 for CMS) showed that the standard curves were not different. Thus, the global equations with the shared parameters were y = 130.1(±2.6)x − 195.3(±12.1) for CSA, y = 125.2(±5.1)x − 372.8(±55.0) for CSB, and y = 1.425(±0.285)x^2^ + 20.97(±7.19)x + 131.6(±37.0) for CMS, and the coefficients of determination (R^2^) were 0.998, 0.993, and 0.996, respectively.

#### 2.2.3. Accuracy and Precision

In order to validate the developed analytical method, the accuracy and precision (in terms of repeatability and intermediate precision) were assessed by analyzing samples at all concentration levels and at three analytical runs. The accuracy was expressed as % standard error from the nominal value. Repeatability is referred to as the precision under the same operating conditions over a short internal of time, whereas intermediate precision refers to the variations between different analytical days. Both terms of precision were expressed as % relative standard deviation (% RSD). The results are presented in Table 2.

#### 2.2.4. Stability

Autosampler and benchtop stabilities were examined (*n* = 3) at three levels of CS and CMS during 3 h for both studies. All compounds were stable, but it should be noted that the applied methodology did not provide access for assessing the stability of the individual forms of the CMS, thus the total amount of CMS was found to be stable. The results are presented in Table 2.

#### 2.2.5. Robustness

Robustness was examined at three levels by performing deliberate changes (±5%) of the column temperature and the % formic acid of mobile phases. The method was robust as the % RSD was lower than 5%. The results are presented in Table 2.

#### 2.2.6. Limit of Detection and Limit of Quantitation

The limit of detection (LOD) and the limit of quantitation (LOQ) were calculated based on the standard deviation of the response (σ) and the slope of the calibration curve (S) using the following equations: 3.3 σ/S for LOD and 10 σ/S for LOQ. The LOD was found to be 0.29 μg mL^−1^ for CSA and 1.38 μg mL^−1^ for CSB, whereas the LOD values were 0.88 μg mL^−1^ and 4.18 μg mL^−1^, respectively. The LOD and LOQ of CMS were calculated from the linear part of the calibration curve and found to be 1.92 μg mL^−1^ and 5.83 μg mL^−1^, respectively. The results are presented in Table 2.

#### 2.2.7. Carry-Over

A non-detectable amount of the analytes was found in the blank injected samples after the injection of the upper limit of quantitation (ULOQ) for CS and CMS, thus no carry-over effect was observed.

### 2.3. Acidic Hydrolysis of CMS

The understanding of the kinetics of acidic CMS in the presence of sulfuric acid was deemed crucial as it is the method of choice for its determination in biological fluids. It is an indirect method of assessment as the circulating CMS is calculated from the difference:

CMS_circ_ = CS_total_ − CS_before hydrolysis_, (1)

where CS_total_ is the concentration after hydrolysis of CMS to CS and CS_before_ hydrolysis is the circulating concentration due to the endogenous transformation of the prodrug to its active form. Two assumptions were made for Equation (1); that is, (a) CS is not hydrolyzed by the action of sulfuric acid and (b) the conversion of CMS to CS under the conditions used is nearly complete. In order to study the second case, CMS hydrolysis was studied by addition of 0.5 M sulfuric acid in accelerated conditions at temperatures 40 °C, 50 °C, and 60 °C. The nature and the concentration of the acid used were selected as the majority of the reported CMS hydrolysis methods propose these conditions. In order to ensure reliable results, the first assumption was also tested, i.e., the stability of CS in the same conditions. No CS degradation was observed in the second case (the CSA and CSB signal remained stable), indicating that the molecule resists hydrolysis by 0.5 M sulfuric acid at 50 °C and 70 °C (Figure S1).

Pilot experiments showed that CMS was hydrolyzed very quickly during the first 10 min under accelerated conditions. Therefore, injections were performed every 2 min for the first 10 min of the hydrolysis study and, consequently, every 10 min until the end of the experiments. Each experiment stopped after the % remaining CMS decreased to the 50% of the initial CMS amount (t_1/2_). The results showed that CMS degradation did not follow zero-, first-, or second-order models, but probably a combination of them. In order to decipher the reaction order, the concentration of CS as well as its transformed concentration values lnC and 1/C versus time were plotted. The results show that the ln of the % remaining CMS versus time for the first 10 min of each temperature, a straight line, y = ax+ b, was obtained. Thus, the CMS hydrolysis for the first min was modeled using a first-order equation:

C_t_ = C_0_ exp(−kt t) (2)

as its ln transformed form:

lnC_t_ = lnC_0_ − kt t (3)

where C_t_ and C_0_ are the CMS concentrations at time t and zero, respectively, and kt is the first-order rate constant.

**Figure 2:**
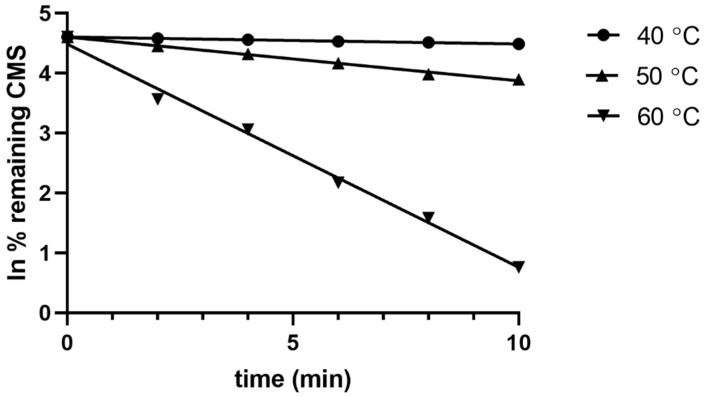
Plots of ln % remaining CMS versus time at the three temperatures for the first 10 min of the CMS degradation in the presence of 0.5 M sulfuric acid. The corresponding linear equations were as follows: y = −0.0116x + 4.6053 (R^2^ = 0.9976) at 40 °C, y = −0.0729x + 4.6043 (R^2^ = 0.9953) at 50 °C, and y = −0.372x + 4.4849 (R^2^ = 0.9891) at 60 °C.

The linearity of the constructed plots at different temperatures reflected the dependence of the CMS hydrolysis constant k on temperature, as described by the Arrhenius kinetics theory. The equations were as follows: y = −0.0116(±0.0002)x + 4.605(±0.001) with R^2^ = 0.9976 at 40 °C, y = −0.072(±0.002)x + 4.60 (±0.01) with R^2^ = 0.9953 at 50 °C, and y = −0.37 (±0.01)x + 4.48 (±0.08) with R^2^ = 0.9891 at 60 °C. The plots for each temperature are presented in Figure 2.

The rate constants of the three temperatures were equal to the slope a of the corresponding linear equations (Equation (3)). In order to model the dependence of the reaction constant against temperature, the activation energy and the rate constants at different temperatures were calculated using the Arrhenius plot and the relevant equation:

lnk = −Ea /RT + lnA(4)

where k is the rate constant of the hydrolysis reaction, Ea is the activation energy (J mol^−1^), R is the universal gas constant (8.314 J mol^−1^K^−1^), T is the temperature (in kelvin), and A is the Arrhenius pre-exponential factor, with a constant for each reaction. The results showed that a linear relationship could be established between lnk and 1/T, showing an R^2^ = 0.996. The activation energy was calculated to be 148,670 J mol^−1^. The Arrhenius plot and the corresponding equation describing the hydrolysis model for the first 10 min of the reaction are shown in Supplementary A (Figure S2).

**Figure 3:**
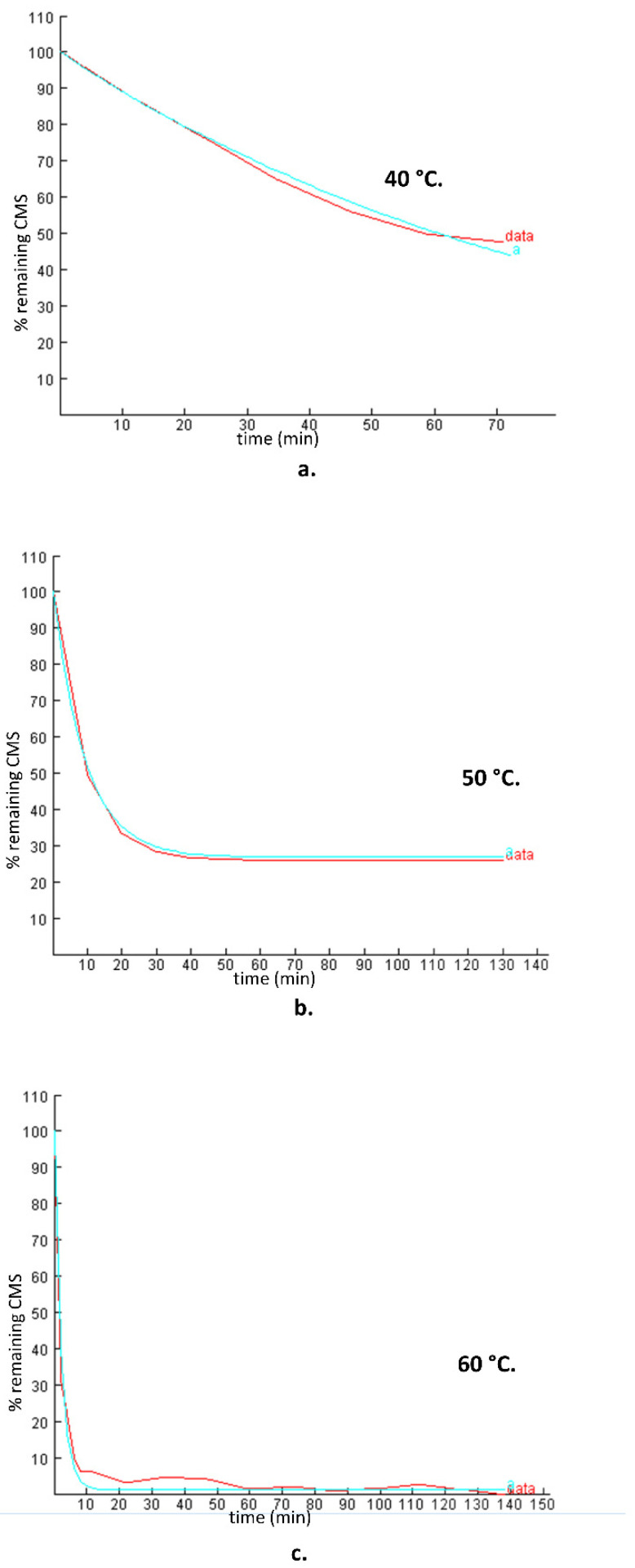
Experimental plots (% remaining CMS versus time) of the CMS degradation in the presence of 0.5 M sulfuric acid (data) and the theoretical models (**a**) simulated by Tenua—the kinetics simulator for Java 2.0—at temperatures of (**a**) 40 °C, (**b**) 50 °C, and (**c**) 60 °C with rate constants of 0.0146 min^−1^, 0.0709 min^−1^, and 0.4485 min^−1^, respectively. The red line represents the experimental data, whereas the blue line represents the theoretical model.

**Figure 4:**
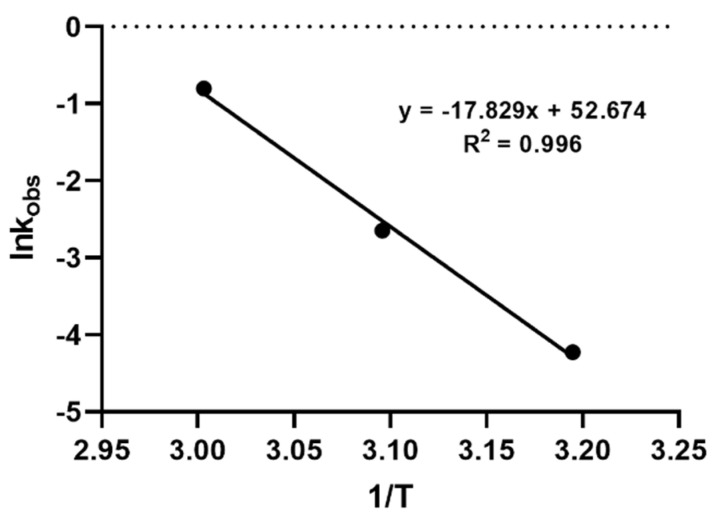
Arrhenius plot for CMS degradation in the presence of 0.5 M sulfuric acid at temperatures 40 °C, 50 °C, and 60 °C.

In order to further explore the hydrolysis kinetics, a computer-based nonlinear least-squares regression approach was employed. Tenua—the kinetics simulator for Java 2.1 (http://bililite.com/tenua/)—is a kinetics program that simulates chemical reactions by fitting suitable differential equations to the experimental data and parameters. Therefore, the data of the above-mentioned hydrolysis experiments including all the time intervals (0 to 130 min) were considered in order to find the rate constants referring to the whole kinetic model of the CMS hydrolysis. Practically, data as a tab-delimited .txt file, including time and CMS concentration, were imported into Tenua, and the initial variable values startTime (the time of the first injection) and endTime (the end of the experiment) were set according to the corresponding values used for each experiment. Time step (the equal time spaces spanning throughout all the kinetic experiment) and epsilon (accuracy) were set at 2 and 1.0 × 10^−8^, respectively, for all the experiments. Finally, a theoretical model that fitted the experimental data was constructed, affording the rate constant of the reaction. The rate constant k(-1) had a minor impact in the model fitting, as its values were small at each experiment (2.2 × 10^−3^, 3.54 × 10^−3^, and 2.11 × 10^−3^ for the models at 40 °C, 50 °C, and 60 °C, respectively). The experimental and the corresponding theoretical model for each temperature are presented in Figure 3. Employing the Arrhenius plot (Figure 4), the activation energy (Ea) was calculated (148,230 J mol^−1^), which was then utilized to find the rate constants and to calculate the t_1/2_ for the CMS in the presence of 0.5 M sulfuric acid at different temperatures.

Comparing the results obtained using the first-order model for the first 10 min and those obtained employing Tenua, it was observed that the results were in very good agreement. The plots of the theoretical and experimental concentration ersus time at 50 °C and 60 °C (Figure 3b,c) indicate that, for the first min of the hydrolysis, a rapid degradation of CMS was observed. The results showed that a steady stage was reached that was depicted as a plateau to the corresponding diagrams. The remaining concentration depends on the temperature: 50% of CMS remained after hydrolysis at 40 °C, 30% at 50 °C, and <10% at 60 °C.

Hydrolysis of CMS in the presence of sulfuric acid was also performed at 20 °C, as a verification of the results. Injections were performed with 10 min intervals. The plot of the CMS hydrolysis at 20 °C is presented in Supplementary A (Figure S3). It was observed that the rate constant k at that temperature was 0.0004 min^−1^, similar to that calculated by the Arrhenius equation (%E = 0.33) (Table 3).

According to the results, the t_1/2_ at 20 °C was 38.5 h, which proves that CMS hydrolysis at room temperature by the addition of 0.5 M sulfuric acid for 1 h was not complete, as previously described. A partial CMS hydrolysis to CS leads to underestimation of CMS levels in biological fluids (Equation (1)). According to the findings, the conditions for a total hydrolysis are the reaction of 0.5 M sulfuric acid for 10 min at 60 °C, when the plateau was reached.

### 2.4. Application in Plasma Samples

Application of the method to spiked plasma samples with CMS was also performed in order to verify the obtained results. Detection of CMS in plasma was not facile in water owing to the existence of interferences by plasma substances (matrix effect). CMS at 20 μg mL^−1^ afforded a low intensity chromatographic peak, in contrast with the same concentration in water. However, this was not deemed as a restriction because it was feasible to study the formation of the hydrolysis products, i.e., CSA and CSB. No matrix effect was detected in CS measurement, as the areas of the two colistin forms were similar (>95%) in plasma and water (Figure S4).

**Figure 5:**
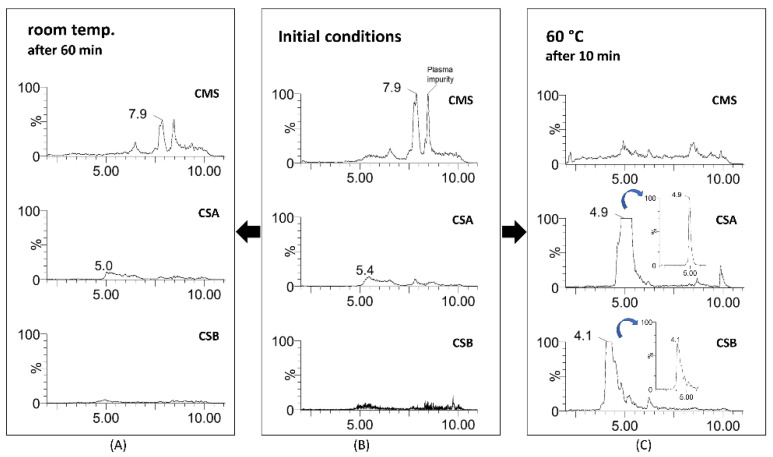
Detection of CMS, CSA, and CSB at initial conditions (room temperature) (**B**) and after hydrolysis with 0.5 M sulfuric acid at 20 °C after 60 min (**A**) and at 60 °C after 10 min (**C**) in plasma samples. CS peaks appeared at 70 °C (CSA at 4.9 min and CSB at 4.1 min) after the total hydrolysis of CMS. The y axis was kept the same in all figures except in the inserts, where it has been normalized.

Plasma samples of 50 μL were spiked with CMS in order to reach concentrations at the 20 μg mL^−1^ and 60 μg mL^−1^ levels. These levels were selected because they have been considered as the concentrations in patient plasma assuming two administratios schemes for CMS i.e., 3 ΜIU for routine dosing and 6 ΜIU as the loading dose [27,28]. In order to evaluate whether the hydrolysis of CMS to the active forms, CSA and CSB, was performed completely, the indirect measurement methodology described in the literature, i.e., 0.5 M sulfuric acid at room temperature, was evaluated. After 1 h at room temperature, no CSA and CSB chromatographic peaks were observed, whereas CMS was detected at 7.9 min, indicating that the hydrolysis of CMS was not completed (Figure 5). The peak appearing at 8.4 min is due to a plasma impurity.

Performing the hydrolysis with sulfuric acid at 60 °C, the results were different. After 10 min, no CMS remained and CS peaks were detected. CSA and CSB were eluted at 4.9 min and 4.1 min, respectively (Figure 5). The experiment was performed at the two aforementioned CMS concentrations: 20 μg mL^−1^ and 60 μg mL^−1^. The ratio of CSA and CSB after CMS hydrolysis was different than the corresponding in the CS reference standard, i.e., the CSA peak was larger, but the total CS remained the same.

Although the hydrolysis of CMS to the active form of CS in vivo is necessary for the effectiveness of the drug, the hydrolysis in pharmaceutical formulations before the administration is undesirable, because it increases the toxicity. The currently developed and validated method could be used for the quality control of CMS products by detecting possible hydrolysis products, i.e., CSA and CSB. Furthermore, the method provides a quick estimation of the CMS content in pharmaceutical products, supporting the microbiological method that is currently used that actually measures the activity of CMS, but is considered of higher uncertainty.

## 3. Materials and Methods

### 3.1. Chemical and Reagents

CS (mixture of colistin sulfate A and B, purity 92.2%) was purchased from Analytical Standard Solutions (Saint Jean d’Illac, France) and CMS was obtained from the local market. Acetonitrile and formic acid were purchased from Carlo Erba reagents (Val de Reuil Cedex, France), whereas methanol was obtained from Fisher Scientific (Loughborough, UK). All solvents are of LC-MS grade of purity. Sulfuric acid, sodium hydroxide, and trifluoroacetic acid (TFA) were obtained from Sigma Aldrich (Steinheim, Germany). Ultra-pure water was produced by a Millipore Direct-Q System (Molsheim, France).

### 3.2. Liquid Chromatography

An Acquity UPLCTM system (Waters Corp., Milford, MA, USA) equipped with a binary solvent manager system and a sample manager thermostatically controlled at 4 °C was used. The column temperature was maintained at 29 °C throughout all experiments. Chromatographic separation was performed on a Waters Acquity BEH C_8_ (2.1 × 100 mm, 1.7 μm, Milford, CT, USA) analytical column. The mobile phase consisted of 0.1% aqueous formic acid (solvent A) and acetonitrile with 0.1% formic acid (solvent B). The gradient elution program was as follows: from 15% B to 27% in 6.10 min; from 27% to 80% in 1.4 min; 0.5 min at 80%; and from 80% to 15% in 0.1 min. The flow rate was 0.15 mL min^−1^ and the total analysis time was 11 min. The full loop injection mode was selected using a 5 μL loop.

### 3.3. Mass Spectrometry

A Waters quadrupole time-of-flight mass spectrometer (QToF Premier, Milford, CT, USA) equipped with an electrospray ionization (ESI) interface was used. The mass spectrometer was operated in positive ion mode and a full scan mode over the range of 1000–1500 Da was selected. The electrospray voltage, the sample cone voltage, and the extraction cone voltage were kept at 3.0 kV, 80 V, and 5.0 V, respectively. The MCP plates were operated at 1950 V. Nitrogen was used as the desolvation gas and was set at 900 L h^−1^ and 400 °C. The source temperature was 120 °C. The TOF analyzer was operated in the V optics mode, affording a resolution of 9000. The scan time was 0.2 s with employing an inter scan delay of 0.02 s. The MassLynx (version 4.1 SCN 872, Waters Corp., Milford, MA, USA) software was used for instrument control, data acquisition, and processing.

### 3.4. Preparation of Standard Solutions

Stock solutions of CS and CMS were prepared in methanol at a concentration of 1 mg mL^−1^ and were stored at −30 °C in order to avoid sample degradation. Standard working solutions of analytes were prepared at 100 μg mL^−1^ by dilution of the stock solutions with methanol.

Calibration standards for CS and CMS were prepared in water at concentration levels of 2.0, 6.0, 8.0, 12.0, 16.0, 20.0, and 22.0 μg mL^−1^. The calibration levels corresponded to 1.806, 2.408, 3.612, 4.816, 6.02, and 6.622 μg mL^−1^ for CSA and 4.194, 5.592, 8.388, 11.184, 13.98, and 15.378 μg mL^−1^ for CSB. The 16.0 mg mL^−1^ corresponds to 100% nominal value (10 MIU), whereas the four flanking levels correspond to 60, 80, 120, and 140% of the aforementioned value.

### 3.5. Assay Validation

The assay was validated in terms of linearity, precision and accuracy (intra- and inter-day), stability, robustness, limit of quantitation (LOQ), and limit of detection (LOD) according to the ICH Q2(R1) analytical procedure guidance (https://www.ema.europa.eu/en/documents/scientific-guideline/ich-q−2-r1-validation-analytical-procedures-text-methodology-step-5_en.pdf).

### 3.6. Acidic Hydrolysis of CMS

In the study, 50 μL of CMS samples was acidified with 12.5 μL of 0.5 M sulfuric acid and the kinetics of drug degradation were monitored at 2 min intervals for the first 10 min and then at 10 min intervals. The reaction was stopped and neutralized by the addition of 12.5 μL of 1 M sodium hydroxide solution and the samples were injected into the UPLC-QToF. The acidic hydrolysis was tested at three temperatures: 40 °C, 50 °C, and 60 °C.

### 3.7. Application to Plasma Samples

In the study, 50 μL of drug free human plasma samples was spiked with CMS in order to obtain a final concentration of 20 μg mL^−1^, followed by acidic hydrolysis, as described above. The protein precipitation was performed using the method described by Jansson et al. [20] with minor modifications. The samples were precipitated with 200 μL acetonitrile containing 0.1% TFA (*v*/*v*), vortexed for 10 s, and centrifuged at 10,000 rpm for 5 min. The supernatant was evaporated under an N_2_ stream. The dry residue was reconstituted with 50 μL H_2_O and, after vortexing, the samples were transferred to 200 μL inserts placed in appropriate screw-capped autosampler vials.

## 4. Conclusions

CMS is widely administrated for the treatment of “superbugs”, but the dosage regimen is still unclear. Thus, several methodologies have been reported for the measurement of CMS in biological material for shedding light to its pharmacokinetics. However, because of the complexity of CMS, the reported methodologies are based on the indirect determination of CMS after its hydrolysis to CS. In the presented study, the Arrhenius equation was assessed in order to find the conditions that lead to a complete hydrolysis of CMS to CS. According to the results, the indirect determination of CMS with the previous reported hydrolysis methodologies led to the underestimation of its levels in plasma, as the hydrolysis to CS was incomplete. The conditions proposed for a complete reaction involved the addition of 0.5 M sulfuric acid at 60 °C. After the application of the method in plasma samples, no CMS was detected, contrary to the previously reported conditions where a significant amount of CMS remained unhydrolyzed. The UPLC-MS methodology that was developed and validated for the study of the CMS kinetics could also be applied for the simultaneous quantitation of CMS and CS in pharmaceutical products as a quality control procedure.

It is evident that accurate measurement of CMS in plasma is of great importance, as inaccurate quantitation will lead to erroneous pharmacokinetic results, and thus to inappropriate dosage regimen and ultimately to acceleration of the neuro- and nephro-drug toxicity. The goal is to find a dosage regimen of CMS that minimizes the toxicity and increases the therapeutic capacity, maintaining the benefit–risk balance, which can be achieved starting with an accurate measurement of CMS in patient plasma. As a next step, the proposed methodology could be validated in plasma samples.

## Figures and Tables

**Table 1:** The reported methods for the hydrolysis of colistimethate sodium (CMS) to colistin (CS) in plasma.

Authors	Acid	Concentration (M)	Reaction Time (min)	Temp (°C)
Li et al. [19]	sulfuric	0.08–0.1	10	room
Jansson et al. [20]	sulfuric	1	15–20	room
Gobin et al. [21]	sulfuric	0.5	60	room
Gikas et al. [18]	sulfuric	0.5	60	room
Mercier et al. [22]	sulfuric	1	30	room
Zhao et al. [15]	sulfuric	0.5	15	room
Bihan et al. [16]	sulfuric	1	60	room

**Table 2:** Accuracy, repeatability, intermediate precision, stability (autosampler and benchtop), robustness, limit of detection (LOD), and limit of quantitation (LOQ) values calculated for CSA, CSB, and CMS. RSD, relative standard deviation.

Analyte (μg mL^−1^)	Accuracy * (*n* = 3, %Error)	Repeatability * (*n* = 3, %RSD)	Intermediate Precision (%RSD)	Autosampler Stability (*n* = 3, %RSD)	Benchtop Stability (*n* = 3, %RSD)	Robustness (*n* = 3, %RSD)
CSA						
1.81	<6.15	<3.71	1.86	2.78	3.45	<4.45
2.41	<1.41	<2.55	1.28			
3.61	<11.05	<5.61	9.07	1.15	3.12	<3.12
4.82	<4.71	<1.73	4.23			
6.02	<2.26	<5.14	3.44			
6.62	<3.21	<5.42	3.58	1.96	2.95	<2.14
CSB						
4.19	<1.71	<5.71	5.16	2.53	3.56	<4.27
5.59	<1.68	<3.80	2.48			
8.39	<2.02	<4.39	5.59	1.84	3.78	<2.95
11.18	<5.33	<2.55	2.13			
13.98	<5.07	<3.82	2.93			
15.38	<4.61	<4.85	3.71	1.69	3.17	<1.97
CMS						
2	<11.01	<5.41	5.96			
6	<13.21	<5.71	5.74	2.35	7.54	<4.12
8	<7.91	<4.27	5.06			
12	<4.79	<4.54	5.98	1.87	5.58	<4.05
16	<4.71	<3.84	2.30			
20	<1.53	<1.85	1.29			
22	<0.40	<0.53	0.37	2.56	5.87	<3.94
	LOD (μg mL^−1^)		LOQ (μg mL^−1^)			
CSA	0.29		0.88			
CSB	1.38		4.18			
CMS	1.92		5.83			

* The maximum absolute values observed at the three analytical runs in three days.

**Table 3:** Rate constant kt and t_1/2_ values for CMS degradation in the presence of 0.5 M sulfuric acid at different temperatures (20 °C, 40 °C, 50 °C, and 60 °C), as calculated by Tenua.

Temp (°C)	kt (min^−1^)	t_1/2_ (0.693/K) (min)
20 *	0.0003	2310.00
40 **	0.0146	47.47
50 **	0.0709	9.78
60 **	0.4485	1.55

* Used for verification of the Arrhenius analysis. ** Used for the construction of the Arrhenius plot.

## Data Availability

The data presented in this study are available in this article and supplementary material.

## Supplementary Materials

The following are available online. Figure S1: Plots of CSA and CSB areas versus time after addition of 0.5 M sulfuric acid at 50 and 70 °C. Both CSA and CSB are stable during the experiment, Figure S2: Arrhenius plot for the degradation of CMS in presence of 0.5 M sulfuric acid at temperatures 40 °C, 50 °C and 60 °C for the first 10 min, Figure S3: Plot of ln % remaining CMS versus time of the CMS degradation in the presence of 0.5 M sulfuric acid at 20 °C. The corresponding linear equation was y = −0.0004x + 7.5189, Figure S4: The two chromatographic peaks of CS in water; (a) CSA, (b) CSB at retention time 4.90 min and 3.88 with areas 43 and 211, respectively, and in plasma; (c) CSA, (d) CSB at retention time 4.95 min and 3.90 min with areas 45 and 212, respectively.

## Sample Availability

Samples of the compounds are not available from the authors.
