# Supplementary material for: Colistimethate Acidic Hydrolysis Revisited: Arrhenius Equation Modeling Using UPLC-QToF MS
Source: Molecules. 2021 Jan 16;26(2):447. doi: 10.3390/molecules26020447 (PMC7830259; doi:10.3390/molecules26020447)
Supplement: Supplementary file 1 [file molecules-26-00447-s001.pdf]

Supplementary A

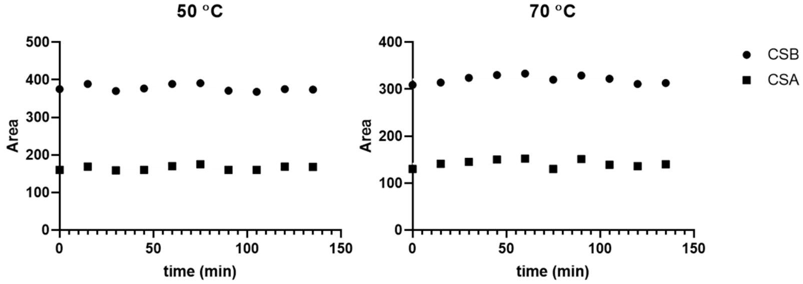

Figure S1. Plots of CSA and CSB areas versus time after addition of 0.5 M sulfuric acid at 50 and 70 °C. Both CSA and CSB are stable during the experiment.

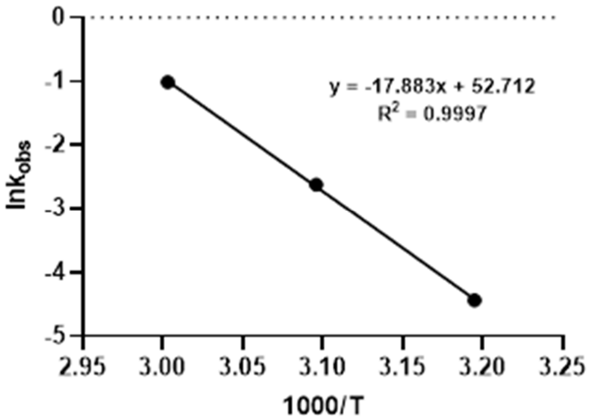

Figure S2. Arrhenius plot for the degradation of CMS in presence of 0.5 M sulfuric acid at temperatures 40 °C, 50 °C and 60 °C for the first 10 min.

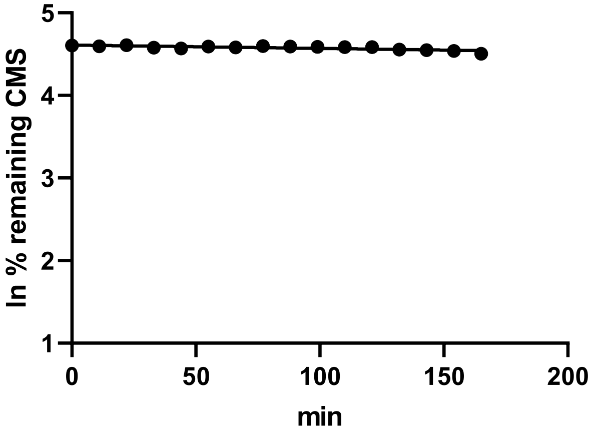

Figure S3. Plot of ln % remaining CMS versus time of the CMS degradation in the presence of 0.5 M sulfuric acid at 20 °C. The corresponding linear equation was y = -0.0004x + 7.5189.

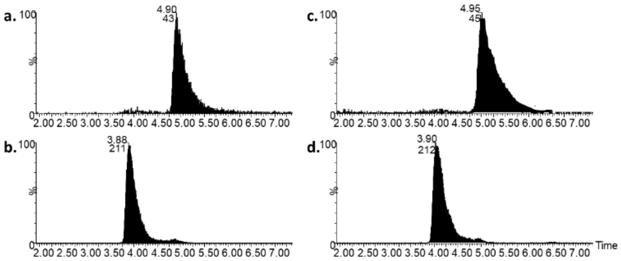

Figure S4. The two chromatographic peaks of CS in water; (a) CSA, (b) CSB at retention time 4.90 min and 3.88 with areas 43 and 211, respectively, and in plasma; (c) CSA, (d) CSB at retention time 4.95 min and 3.90 min with areas 45 and 212, respectively.
